# Editorial: Diabetic wound: multifaceted mechanisms and future of diabetic wound healing, volume II

**DOI:** 10.3389/fendo.2025.1650648

**Published:** 2025-07-11

**Authors:** Shiying Shao, Johnson Boey, Xingwu Ran

**Affiliations:** ^1^ Division of Endocrinology, Department of Internal Medicine, Tongji Hospital, Tongji Medical College, Huazhong University of Science and Technology, Wuhan, China; ^2^ Hubei Clinical Medical Research Center for Endocrinology and Metabolic Diseases, Hubei, China; ^3^ Branch of National Clinical Research Center for Metabolic Diseases, Hubei, China; ^4^ Department of Podiatry, National University Hospital Singapore, Singapore, Singapore; ^5^ Department of Endocrinology and Metabolism, West China Hospital of Sichuan University, Chengdu, China

**Keywords:** mesenchymal stem cells, non-coding RNAs, diabetic foot infection, diabetic foot ulcer, fibrous proteins, machine learning, transtibial transport

Diabetic foot ulcer (DFU) represents a severe complication of diabetes, which will develop in an estimated 15%-25% of diabetic patients globally during their lifetime (1). DFU is associated with significant disability and mortality rates, with reported annual mortality rate of 11% and mortality rate among those who undergo amputation surgery exceeding 22% (2). The management of DFU continues to pose substantial clinical challenges. Six recent studies published in “Diabetic wound: multifaceted mechanisms and future of diabetic wound healing, volume II” shed light on innovative therapeutic strategies that may help to transform patient outcomes.

Mesenchymal stem cells (MSCs) have been recently widely investigated in the field of wound management due to their powerful regenerative capabilities and immunomodulatory properties (3, 4). Of note, the regenerative effects of MSCs are mainly attributed to extracellular vesicles (EVs). A meta-analysis by Yue et al. on MSC-derived small EVs (MSC-sEVs) included 21 preclinical studies, which reported that MSC-sEVs could significantly enhance wound closure, angiogenesis, collagen deposition, and anti-inflammatory responses in type 2 diabetic models. Notably, local injection may be the optimal route of administration for EVs due to their rapid systemic clearance. Despite these promising results, the clinical translation of sEVs faces several hurdles, such as standardization of isolation methods, scalability, and safety concerns. Rigorous randomized controlled trials (RCTs) to validate these findings in humans are needed.

Complementing this, Aghayants et al. provide a comprehensive review of non-coding RNAs (ncRNAs) as regulators of diabetic wound healing. Their work elucidates the roles of microRNAs (miRNAs), long non-coding RNAs (lncRNAs), and circular RNAs (circRNAs) in modulating inflammation, angiogenesis, and tissue remodeling. The study indicated that the recent advancement in nanomedicine may contribute to the therapeutic promise of ncRNA-based interventions by improving the stability and targeting precision of exogenous ncRNAs.

Fibrous proteins, particularly collagen and elastin, serve as key structural components of the extracellular matrix (ECM), which play a critical role in wound repair and tissue regeneration (5). Yan et al.’s review on fibrous proteins underscores the potential of biomaterials to revolutionize diabetic wound care. The authors emphasize that advanced wound dressings incorporating fibrous proteins offer bioactive properties that go beyond traditional passive dressings. These materials not only provide structural support but also modulate cellular behavior, promote angiogenesis, and reduce oxidative stress. However, most of the literature cited in this review are animal experimental articles and clinical evidences are limited.

Ni et al. offered a therapeutic potential of a traditional Chinese medicine (TCM) formulation, Danggui Sini decoction (DSD). DSD is a TCM prescription with a long history of use for blood deficiency and cold coagulation syndromes. Using network pharmacology and molecular docking, the authors identify that the major molecular pathways of DSD may be mediated by AGE-RAGE and PI3K-AKT signaling. Their meta-analysis of six RCTs with a total of 444 patients further validates DSD’s efficacy on the improvement of wound repair and ankle-brachial index.

Interestingly, Guan et al. investigate machine learning (ML)’s applications in DFU, emphasizing its capacity to analyze complex datasets from imaging, biomarkers, and clinical biomechanics. ML algorithms, such as convolutional neural networks (CNNs) and XGBoost, have demonstrated their remarkable accuracy in detecting early DFU (e.g., via thermography), predicting ulcer healing, and stratifying amputation risks. The authors also discuss challenges of ML, including data quality and model interpretability, and propose the potential solutions like SHAP (SHapley Additive exPlanations) to elucidate model decision-making processes. With ongoing technological advancements, the application of ML presents unprecedented opportunities to redefine the diagnostic and therapeutic strategies of diabetic foot care.

Furthermore, it has been predicted that nearly 50% of patients with DFU suffer from foot infections (6). Boey et al. present a compelling case report on the use of transtibial transport (TTT) to manage thromboembolic (TE) events following surgical intervention for necrotizing soft tissue infection (NSTI) in a 70-year-old patient with poorly controlled diabetes. TTT is a technique based on distraction osteogenesis, which has been widely applied in ischemic foot disease. The clinical effect of TTT on the resolution of TE events remains unclear, which needs more investigations.

This editorial integrates cutting-edge findings focusing on diagnostic and therapeutic strategies for DFU. Original research and review articles published in this Research Topic provide new perspectives on treatment trends for DFU. We sincerely acknowledge the collaborative efforts of all contributors and reviewers, which have facilitated innovation within the field. Looking ahead, we are committed to translating these research outcomes into patient-centered practice protocols to improve clinical outcomes.
